# ﻿Morphological characteristics and phylogenetic evidence reveal two new species and the first report of *Comoclathris* (Pleosporaceae, Pleosporales) on dicotyledonous plants from China

**DOI:** 10.3897/mycokeys.101.113040

**Published:** 2024-01-12

**Authors:** Rong Xu, Wenxin Su, Yang Wang, Shangqing Tian, Yu Li, Chayanard Phukhamsakda

**Affiliations:** 1 School of Food Science and Engineering, Yangzhou University, Yangzhou 225127, China Jilin Agricultural University Changchun China; 2 Internationally Cooperative Research Center of China for New Germplasm Breeding of Edible Mushroom, Jilin Agricultural University, Changchun 130118, China Yangzhou University Yangzhou China; 3 College of Plant Protection, Shenyang Agricultural University, Shenyang, 110866, China Shenyang Agricultural University Shenyang China; 4 Center of Excellence in Fungal Research, Mae Fah Luang University, Chiang Rai 57100, Thailand Mae Fah Luang University Chiang Rai Thailand

**Keywords:** Ascomycota, *Clematis*, new species, saprobes, taxonomy, *Xanthocerassorbifolium*

## Abstract

Two novel *Comoclathris* species were identified from dicotyledonous plants (*Clematis* sp. and *Xanthocerassorbifolium*) in China. The results were supported by morphological characters and Maximum Likelihood (ML) and Bayesian Inference (BI) analyses. Multi-gene phylogenetic analyses of the ITS, LSU, SSU and *rpb*2 sequences revealed two new species *Comoclathrisclematidis* and *C.xanthoceratis*, which are phylogenetically distinct. The new species are phylogenetically closely related to *C.arrhenatheri.* However, they are distinguishable from *C.arrhenatheri* by having comparatively larger asci and ascospores. This study improves our knowledge of *Comoclathris* as no species has been previously described from China. This suggests such taxa may be rare and it is likely that new taxa will be discovered from hosts and environments that have not yet been extensively investigated.

## ﻿Introduction

Clements (1909) introduced the genus *Comoclathris* with *C.lanata* Clem as the type species. The species was originally assigned to the Diademaceae, based on having ascomata with flat circular lid-like opening (Shoemaker and Babcock 1992). Previously, *Comoclathris* was considered a synonym of *Platyspora* (Ariyawansa et al. 2014) and *Comoclathris* has been associated with an asexual morph resembling *Alternaria*-like (Simmons 1967); thus, the genus was temporarily referred to Pleosporaceae, based to these morphological characteristics (Zhang et al. 2012; Woudenberg et al. 2013). Two strains of *Comoclathriscompressa* (CBS 157.53 and CBS 156.53) were treated as representative sequences which formed a well-supported clade within the family Pleosporaceae (Ariyawansa et al. 2014). Subsequently, *Comoclathris* was placed into Pleosporaceae, based on phylogenetic evidence coupled with morphological characteristics (Ariyawansa et al. 2015; Thambugala et al. 2017; Wijayawardene et al. 2017; Wanasinghe et al. 2018).

*Comoclathris* can be distinguished from *Pleospora*, *Pleoseptum* and *Clathrospora* by its applanate and dark reddish-brown muriform ascospores with a single longitudinal septum and ascomata with circular lid-like opening (versus two or more rows of longitudinal septa of *Clathrospora* species) (Shoemaker and Babcock 1992; Zhang et al. 2012; Ariyawansa et al. 2014, 2015). Thirty-eight epithets have been recorded as *Comoclathris* in Species Fungorum (2023); however, most lack molecular data, including the type species *C.lanata*. *Comoclathris* has been found from America, Antarctica, Argentina, Austria, Bulgaria, Canada, Central Asia, Finland, Greece, India, Iran, Iraq, Italy, Netherlands, Norway, Pakistan, Portugal, Romania, Russia, Spain, Sweden, Switzerland, Syria, Tunisia, Turkey, Ukraine and Yugoslavia (Ahmad 1978; Shoemaker and Babcock 1992; Chlebicki 2002; Checa 2004; Pande 2008; Woudenberg et al. 2013; Eriksson 2014; Thambugala et al. 2017; Hongsanan et al. 2020). Most *Comoclathris* species are saprobes, with recent reports from Italy (Hyde et al. 2016; Wanasinghe et al. 2018; Brahmanage et al. 2020).

The aim of this study was to explore the diversity of *Comoclathris* species from dicotyledonous plants in China. Two new *Comoclathris* species (*C.clematidis* and *C.xanthoceratis*) from Jilin and Yunnan Provinces, China are described. The morphology was compared to other *Comoclathris* species. Maximum Likelihood and Bayesian Inference phylogenetic analyses were performed to confirm the taxonomic position of the isolates using ITS, LSU, SSU and *rpb*2 datasets. The results improve our understanding of the occurrence and distribution of *Comoclathris* species from China, thus expanding the knowledge of fungal biodiversity. This is also the first report of *Comoclathris* on dicotyledonous plants in China.

## ﻿Materials and methods

### ﻿Sample collection, morphological study and isolation

Dried wood samples were collected from Jilin (Temperate zone, 43°10′N, 124°20′E) and Yunnan Provinces (Subtropical region, 25°23′N, 102°42′E) in China. The samples were transferred to the laboratory in plastic bags with labels indicating the details of the collection. The characteristics of specimens were observed using a Zeiss Stemi 2000C stereomicroscope, equipped with a Leica DFC450C digital camera (Leica, Germany). Morphological characteristics of ascomata (n = 5), peridium (n = 10), hamathecium (n = 20), asci (n = 20), ascospores (n = 40) and other microscopic characteristics associated with ascomata were documented using a Zeiss AX10 microscope, equipped with an Axiocam 506 digital camera (ZEISS, Germany). The ZEN 3.4 application (blue edition) was used for microscopic measurements (ZEISS, Germany). The photos were edited using Adobe Photoshop CC2020 (Adobe Systems, USA).

Single spore isolation was used to obtain pure cultures (Senanayake et al. 2020) and germinated spores were cultured at 25 °C on potato dextrose agar (PDA). Type specimens were deposited in the Herbarium of Mycology, Jilin Agricultural University (HMJAU), Changchun, China and isotypes were deposited in Mae Fah Luang University (MFLU) Herbarium, Chiang Rai, Thailand. Ex-type cultures were deposited in the International Cooperation Research Center of China for New Germplasm Breeding of Edible Mushrooms Culture Collection (CCMJ). The new taxa were registered in MycoBank (Crous et al. 2004).

### ﻿DNA extraction, PCR amplification and sequencing

Pure mycelia were harvested after two weeks of incubation at 25 °C on PDA. The internal transcribed spacer regions (ITS), large subunit (LSU), small subunit (SSU) and RNA polymerase II second-largest subunit (rpb2) were amplified by polymerase chain reaction (PCR) using ITS5/ITS4, NS1/NS4 (White et al. 1990), LR0R/LR5 (Vilgalys and Hester 1990) and fRPB2-5F/fRPB2-7cR (Liu et al. 1999) primers, respectively. The amplification reactions and conditions for ITS, LSU and SSU were performed using the conditions described by Xu et al. (2022). The amplification conditions for *rpb*2 annealing conditions were different: 94 °C for 5 min, then 35 cycles of denaturation at 94 °C for 30 s, annealing at 56 °C for 45 s, elongation at 72 °C for 90 s and a final extension at 72 °C for 10 min. The amplification reactions were performed using 20 μl PCR mixtures containing 9 μl ddH_2_O, 10 μl of 2× EsTaq MasterMix (Dye), 0.4 μl (200 ng/µl) of DNA template and 0.3 μl of 2 μmol/μl of forward and reverse primers. The PCR products were verified on 1% agarose electrophoresis gels stained with 0.5 ml of 10,000X standard DNA dye (Biotium, United States). Purification and sequencing of amplified PCR fragments were performed by Sangon Biotech Co, Shanghai, China.

### ﻿Sequencing and sequence alignment

Sequences obtained from this study were searched in the GenBank database (http://blast.ncbi.nlm.nih.gov/) using BLAST. The newly-obtained sequences and data from recent publications (Brahmanage et al. 2020; Crous et al. 2021) were used in the analysis (Table 1). *Neocamarosporiumbetae* (CBS 523.66) and *N.calvescens* (CBS 246.79) were used as the outgroup in the phylogenetic analyses. The sequences were edited using BioEdit v. 7.1.3.0 and aligned with MAFFT v. 7 (Hall 1999; Katoh and Standley 2013). The alignments were trimmed using trimAI v. 1.2 under the gappyout option (Capella-Gutierrez et al. 2009). The datasets were combined using SequenceMatrix v. 1.7.8 (Vaidya et al. 2011). The newly-generated sequence data were deposited in GenBank (Benson et al. 2013).

**Table 1.** Taxa used in the phylogenetic analyses and their corresponding GenBank accession numbers. The ex-type strains are indicated in bold and the newly-generated sequences are shown in cells with light grey shading.

Taxa	Strain	Host/Substrate	Country	GenBank accession numbers				References
				ITS	LSU	SSU	*rpb*2	
*Comoclathrisambigua*	CBS 366.52	–	USA	KY940748	AY787937	–	KT216533	(Woudenberg et al. 2017)
***C.antarctica***	**WA0000074564**	**Soil**	**Antarctica**	**MW040594**	**MW040597**	–	–	(Crous et al. 2021)
*C.arrhenatheri*	MFLUCC 15-0465	*Arrhenatherumelatius*	Italy	KX965737	KY000647	KX986348	KX938346	(Thambugala et al. 2017)
*C.arrhenatheri*	MFLUCC 15-0476	*Dactylisglomerata*	Italy	KY026595	KY000648	KX986349		
***C.clematidis***	**CCMJ 13076**	***Clematis* sp.**	**China**	**OQ534243**	**OQ534239**	**OQ676454**	**OQ547800**	This study
*C.clematidis*	CCMJ 13077	*Clematis* sp.	China	OQ534244	OQ534240	OQ676455	OQ547801	
*C.compressa*	CBS 156.53	*Castilleja miniata*	USA	–	KC584372	KC584630	KC584497	(Woudenberg et al. 2013)
*C.compressa*	CBS 157.53	–	USA	–	MH868679	KC584631	KC584498	
***europaeae***	**MFLU 20-0391**	–	Italy	**MT370396**	**MT370421**	**MT370367**	**MT729650**	(Brahmanage et al. 2020)
***C.flammulae***	**MFLU 20-0397**	***Clematisflammula***	**Italy**	**MT370397**	**MT370422**	**MT370368**	**MT729651**	
*C.flammulae*	MFLU 20-0399	*Coluteaarborescens*	Italy	MT370395	MT370420	MT370366	–	
***C.galatellae***	**MFLUCC 18-0773**	***Galatellavillosa***	**Ukraine**	**MN632549**	**MN632550**	**MN632551**	–	(Hongsanan et al. 2020)
*C.incompta*	CBS 467.76	*Oleaeuropaea*	Greece	–	GU238087	GU238220	KC584504	(Aveskamp et al. 2010)
*C.incompta*	CH-16	*Oleaeuropaea*	Tunisia	KU973716	KU973729	–	–	(Moral et al. 2017)
***C.italica***	**MFLUCC 15-0073**	***Thalictrum* sp.**	Italy	**KX500109**	–	–	–	(Tibpromma et al. 2015,)
***C.lini***	**MFLUCC 14-0968**	***Linum* sp.**	Italy	**KR049218**	**KR049219**	**KT210389**	–	(Nwanasinghe et al. 2015)
*C.lini*	MFLUCC 14-0561	*Ononisspinosa*	Italy	KT591614	KT591615	KT591616	–	
***C.lonicerae***	**MFLU 20-0385**	***Lonicera* sp.**	Italy	**MT370394**	**MT370419**	**MT370365**	**MT729649**	(Brahmanage et al. 2020)
*C.lonicerae*	MFLU 18-1236	*Coluteaarborescens*	Italy	OL744429	OL744433	OL744435	OL771441	
*C.permunda*	MFLUCC 14-0974	*Phleum* sp.	Italy	KY659561	KY659564	KY659568	–	(Vu et al. 2019)
***C.pimpinellae***	**MFLUCC 14-1159**	***Pimpinellatragium***	**Russia**	**KU987665**	**KU987666**	**KU987667**	–	(Li et al. 2016)
***C.rosae***	**MFLU 15-0203**	***Rosacanina***	**Italy**	**MG828876**	**MG828992**	**MG829103**	**MG829249**	(Wanasinghe et al. 2018)
*C.rosae*	MFLU 16-0234	*Rosacanina*	Italy	MG828877	MG828993	MG829104	MG829250	
***C.rosarum***	**MFLUCC 14-0962**	***Rosacanina***	**Italy**	**MG828878**	**MG828994**	**MG829105**	**MG829251**	
***C.rosigena***	**MFLU 16-0229**	***Rosacanina***	**Italy**	**MG828879**	**MG828995**	**MG829106**	**MG829252**	
***C.sedi***	**MFLUCC 13-0763**	***Rosa* sp.**	**Italy**	**KP334717**	**KP334707**	**KP334727**	–	(Ariyawansa,et al. 2014)
*C.sedi*	MFLUCC 13-0817	*Sedum* sp.	Italy	KP334715	KP334705	KP334725	–	
***C.spartii***	**MFLUCC 13-0214**	***Spartiumjunceum***	**Italy**	**KM577159**	**KM577160**	**KM577161**	–	(Cours et al. 2014)
*C.typhicola*	CBS 602.72	–	Netherlands	MH860592	MH872288	–	–	(Vu et al. 2019)
***C.xanthoceratis***	**CCMJ 13078**	***Xanthocerassorbifolium***	**China**	**OQ534245**	**OQ534241**	**OQ676456**	**OQ547802**	This study
*C.xanthoceratis*	CCMJ 13079	*Xanthocerassorbifolium*	China	OQ534246	OQ534242	OQ676457	OQ547803	
*Neocamarosporiumbetae*	CBS 523.66	*Beta vulgaris*	Netherlands	FJ426981	MH870520	EU754080	KT389670	(Aveskamp et al. 2009)
*N.calvescens*	CBS 246.79	*Atriplexcalotheca*	Germany	MH861203	EU754131	EU754032	KC584500	(Vu et al. 2019)

### ﻿Phylogenetic analysis

The phylogenetic analyses were performed using Maximum Likelihood (ML) and Bayesian Inference (BI) methods. RAxML-HPC2 on XSEDE, implemented in the CIPRES web portal (http://www.phylo.org/portal2/), was used for ML analysis, with a rapid bootstrapping algorithm of 1000 replicates (Stamatakis 2014). The suitability of the DNA model was analysed using jModelTest v. 2.1.10 on the CIPRES online portal for posterior probability. The best fit evolutionary models for individual and combined datasets were calculated under the Akaike Information Criterion (AIC) (Nylander 2004) and are as follows: GTR+I+G model for the ITS alignment, K80+I model for the LSU and SSU alignments, GTR+G model for the *rpb*2 alignment and SYM+I+G model for the combined datasets. Bayesian Inference analyses were carried out by using MrBayes v. 3.2.6 on the CIPRES web platform (Ronquist and Huelsenbeck 2003). Tree samples were taken every 1000^th^ generation while Markov chains were run for 15,000,000 generations. Phylogenetic trees were illustrated in FigTree v. 1.4.4 (Rambaut 2018) and altered in Adobe Illustrator CS v. 6. RAxML bootstrap support values greater than or equal to 98% and Bayesian posterior probabilities equal to 1.00 were considered as strong statistical support. The data used in this study were deposited in the Zenodo repository (accession number doi: 10.5281/zenodo.7675986).

## ﻿Results

### ﻿Phylogenetic analyses

**Figure 1.**
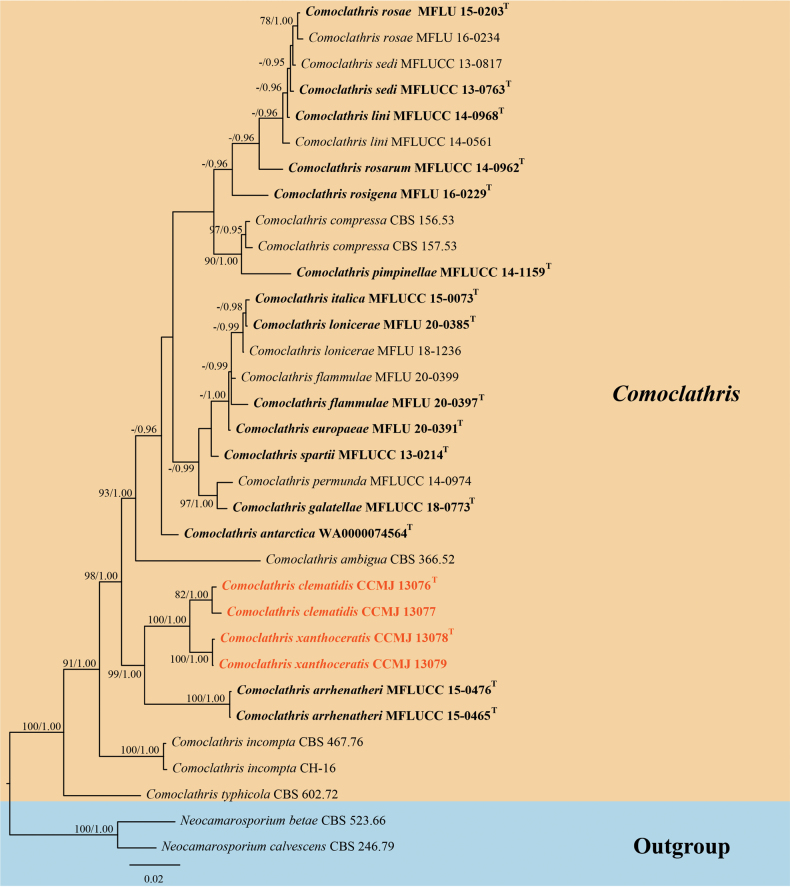
The Bayesian 50% majority-rule consensus phylogram, based on a concatenated ITS, LSU, SSU and *rpb*2 dataset of *Comoclathris.* The tree is rooted with *Neocamarosporiumbetae* (CBS 523.66) and *N.calvescens* (CBS 246.79). RAxML bootstrap support values ≥ 70% (ML, left) and Bayesian posterior probabilities ≥ 0.90 (BPP, right) are shown near the nodes. The new isolates are indicated in orange. The type strains are in bold and labelled with T.

The combined multi-loci (ITS, LSU, SSU and *rpb*2) sequence dataset consisted of 32 taxa and 3,280 characters including gaps (ITS: 1–559 bp, LSU: 560–1,441 bp, SSU: 1,442–2,415 bp and *rpb*2: 2,416–3,280 bp). The best-scoring RAxML tree had a final log-likelihood value of -10805.548630. There were 691 distinct alignment patterns with 26.80% undetermined characters or gaps in the matrix. Estimated base frequencies were as follows: A = 0.254018, C = 0.226589, G = 0.268862, T = 0.250530; substitution rates AC = 2.459854, AG = 4.593060, AT = 1.418628, CG = 1.005203, CT = 7.378387 and GT = 1.000000. The proportion of invariable sites (I) was estimated to be 0.690973 and the gamma distribution shape parameter (α) was estimated to be 0.927322. A total of 4,592 trees were sampled in the BI analysis after the 20% burn-in with a stop value of 0.009967. The ML and BI trees were similar in topology (Fig. 1). Phylogenetic results demonstrated that *Comoclathrisclematidis* and *C.xanthoceratis* formed a distinct lineage and clustered with *C.arrhenatheri* with strong statistical support (98% ML and 1.00 BPP). *Comoclathrisclematidis* (CCMJ13076 and CCMJ 13077) and *C.xanthoceratis* (CCMJ 13078 and CCMJ 13079) formed a closely-related clade with high statistical support (100% ML and 1.00 BPP).

### ﻿Taxonomy

#### ﻿ Comoclathris clematidis

Taxon classificationFungiPleosporalesDiademaceae

R. Xu, Phukhams. & Y. Li sp. nov.

7BD5528C-5C11-55F8-A661-3DC109DBA3FE

847614

Fig. 2

##### Etymology.

Refers to the host genus, *Clematis*.

##### Description.

***Saprobic*** on dried branches of *Clematis* species. **Sexual morph: *Ascomata*** 150–230 × 120–150 μm (*x*– = 176 × 138 μm, n = 5), solitary, scattered or aggregated in small groups, immersed to erumpent, subglobose, elongated, black, without a distinct ostiole. ***Peridium*** 10–20 μm wide at the base, 15–20 μm wide at the sides, comprising thick-walled cells of ***textura angularis***, dark brown to black. ***Hamathecium*** comprising numerous, 1–3.5 μm wide (*x*–= 2.0 μm, n = 20), filamentous, septate, rarely branched pseudoparaphyses, hyaline, embedded in a gelatinous matrix, extending above the asci. ***Asci*** 114–174 × 27–43 μm (*x*– = 140 × 34 μm, n = 20), 8-spored, bitunicate, fissitunicate, cylindrical-clavate, short pedicellate, apically rounded, with an ocular chamber. ***Ascospores*** 22–39 × 8–21 μm (*x*– = 30 × 14 μm, n = 40), 1–2-seriate, partially overlapping, broadly fusiform, initially 3-septate and yellowish, becoming brown, verrucose or echinulate wall, muriform, with 3 transversely septa and a vertical septum in second and third cells, constricted at the septa, with obtuse ends, smooth-walled, surrounded by a thick mucilaginous sheath. **Asexual morph**: Undetermined.

##### Culture characteristics.

Colonies on PDA reaching 40 mm diam. after three weeks at 25 °C. Cultures from above, circular, flat to umbonate, covered with flocculent aerial mycelium, velvety on the surface, greenish-olivaceous, dense, entire edge; reverse black in the middle, green olivaceous radiating outwardly, white mycelium at the edge.

**Figure 2.**
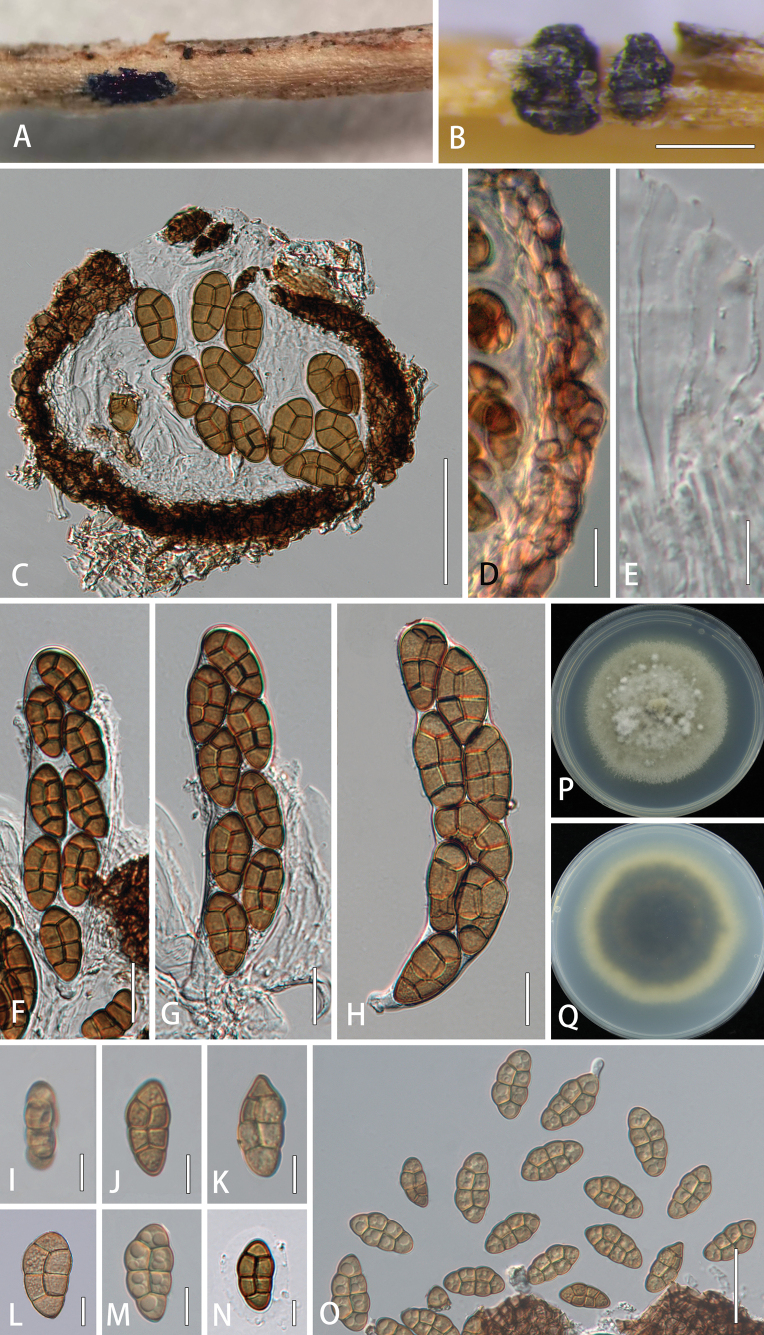
*Comoclathrisclematidis* (HMJAU 64844, holotype) **A, B** appearance of ascomata on host substrate **C** vertical section of ascoma **D** peridium **E** pseudoparaphyses **F–H** asci **I–O** ascospores **P, Q** culture characteristics on PDA after three weeks at 25 °C. Scale bars: 200 µm (**B**); 50 µm (**C**); 20 µm (**E–H, O**); 10 µm (**D, I–N**).

##### Material examined.

China. Yunnan Province, Kunming, on the dead aerial branch of *Clematis* sp. (Ranunculaceae), 24 April 2021, S. Tibpromma, S42, HMJAU 64844 (***holotype***); ex-type, CCMJ 13076; MFLU 23-0384 (isotype), ex-isotype, CCMJ 13077.

##### Notes.

In the phylogenetic analyses, *Comoclathrisclematidis* (CCMJ 13076 and CCMJ 13077) clustered with *C.xanthoceratis* (CCMJ 13078 and CCMJ 13079) with 82% ML and 100 BPP within *Comoclathris* (Fig. 1). *Comoclathrisclematidis* was found on dried stems of *Clematis* species in the subtropical zone of Yunnan Province, China. The majority of *Comoclathris* species are found in temperate regions, but only *C.incompta* (CH-16) has been identified in subtropical regions (Moral et al. 2017). *Comoclathrisclematidis* differs from *C.flammulae* which was also found on *Clematis* by its larger asci (114–174 × 27–43 µm vs. 50–55 × 13–17 µm) and larger ascospores (22–39 × 8–21 µm vs. 16–22 × 10–16 µm). In addition, *C.clematidis* contains fewer transverse septa in ascospores (3 transverse septa vs. 6 transverse septa) (Brahmanage et al. 2020). The new species *Comoclathrisclematidis* is distinguishable from *Comoclathrissedi* which was also isolated from *Clematis* by having larger asci (114–174 × 27–43 µm vs. 80–110 × 16–18 µm), larger ascospores (22–39 × 8–21 µm vs. 19–20 × 8–10 µm) and fewer ascospore septa (3 transverse septa vs. 4–5 transverse septa) (Ariyawansa et al. 2015). The ascomata of *C.clematidis* are immersed to superficial and appear as black spots or convex surfaces, while the ascomata of *C.xanthoceratis* are immersed to semi-immersed and covered with dark brown setae. *Comoclathrisclematidis* has cylindrical-clavate asci and verrucose or echinulate ascospore walls, while *C.xanthoceratis* has clavate asci and smooth-walled ascospores. Both *C.clematidis* and *C.xanthoceratis* have ascospores with 3 transverse septa and 2 vertical septa. In addition, the two species show different culture characteristics and only *C.xanthoceratis* produce ascocarps in the culture. The ITS and *rpb*2 base pair differences between the two species are 0.95% (5/526, no gaps) and 4.69% (34/725, no gaps), respectively.

In the BLASTn search, the *rpb*2 sequence was 89.53% similar to *Comoclathrisarrhenatheri* (MFLUCC 15-0465) with 100% query cover, translating to 89.53% similarity. The LSU sequence was 98.76% similar to *C.permunda* (CBS: 127967) with 99% query cover, translating to 97.77% similarity, while the SSU sequence was 98.58% similar to *C.lini* (MFLUCC 14-0968) with 100% query cover, translating to 98.58% similarity. The ITS region was 97.93% similar to *Comoclathris* sp. (14APR) with 93% query cover, translating to 91.07% similarity. Therefore, *Comoclathrisclematidis* was introduced as a novel species.

#### ﻿ Comoclathris xanthoceratis

Taxon classificationFungiPleosporalesDiademaceae

R. Xu, Phukhams. & Y. Li sp. nov.

7A68C295-6807-5493-86D5-071F1E352E08

847615

Fig. 3

##### Etymology.

Refers to the host genus, *Xanthoceras*.

##### Description.

***Saprobic*** on dried stems of *Xanthocerassorbifolium*. **Sexual morph: *Ascomata*** solitary, scattered or aggregated in small groups, 147–221 × 114–130 μm (*x*– = 187–124 μm, n = 5), immersed to semi-immersed, subglobose, black, elongated, covered with dark brown setae, without a distinct ostiole. ***Peridium*** 13–20 μm wide at the base, 20–32 μm wide at the sides, comprising thick-walled cells of ***textura angularis***, dark brown to black; inner layer composed of thin-walled cells of ***textura angularis***, hyaline. ***Hamathecium*** comprising 1.5–4.0 μm wide, septate, filiform, embedded in a gelatinous matrix, rarely branched pseudoparaphyses, extending above the asci. ***Asci*** 8-spored, bitunicate, fissitunicate, 99–165 × 36–48 μm (*x*– = 127 × 42 μm, n = 20), clavate, short pedicellate, apically rounded, with an ocular chamber. ***Ascospores*** 23–42 × 9–19 μm (*x*– = 37 × 16 μm, n = 40), 1–2-seriate, muriform, broadly fusiform, with 3 transverse septa and a vertical septum in second and third cells, brown to dark brown, with obtuse ends, smooth-walled, surrounded by a thick mucilaginous sheath. **Asexual morph**: Undetermined.

##### Culture characteristics.

Colonies on PDA reaching 30 mm diam. after three weeks at 25 °C. Cultures from above, dense, round, umbonate, wrinkled and folded, papillate with white aerial mycelium, radial edge, orange at the margin; reverse reddish, white mycelium present at the margin.

##### Material examined.

China. Jilin Province, Changchun, on dead stem of *Xanthocerassorbifolium* Bunge (Sapindaceae), 2 July 2022, Rong Xu, XR71, HMJAU 64846 (***holotype***); ex-type, CCMJ 13078; MFLU 23-0385 (isotype), ex-isotype, CCMJ 13079.

##### Notes.

*Comoclathrisxanthoceratis* (CCMJ 13078 and CCMJ 13079) is closely related to *C.clematidis* (CCMJ 13076 and CCMJ 13077) (100% ML and 1.00 BPP). The two species are phylogenetically closely related to *C.arrhenatheri* (MFLUCC 15-0465). However, there are distinct differences in morphology (Thambugala et al. 2017). The asci of *C.arrhenatheri* are smaller than *C.clematidis* and *C.xanthoceratis* (*C.arrhenatheri* vs. *C.clematidis* vs. *C.xanthoceratis*: 70–95 × 18.5–25 vs. 114–174 × 27–43 vs. 99–165 × 36–48 μm, respectively). *Comoclathrisarrhenatheri* have ascospores with 4 transverse septa and 2–3 vertical septa, while *C.clematidis* and *C.xanthoceratis* only have 3 transverse septa and 2 vertical septa. Additionally, the ascospores of *C.arrhenatheri* are shorter than *C.clematidis* and *C.xanthoceratis* (*C.arrhenatheri* vs. *C.clematidis* vs. *C.xanthoceratis*: 16.5–22 × 7.7–10.2 vs. 22–39 × 8–21 vs. 23–42 × 9–19 µm).

A pairwise comparison of the ITS region between *C.xanthoceratis* and *C.arrhenatheri* demonstrated 8.95% (46/514, no gaps) base-pairs difference, while there were 74 base-pair difference in the *rpb*2 gene (10.2%, no gaps). Hence, *C.xanthoceratis* is introduced as a new species, based on morphological and nucleotide differences. This is also the first report of *Comoclathris* species found on *Xanthocerassorbifolium*.

**Figure 3.**
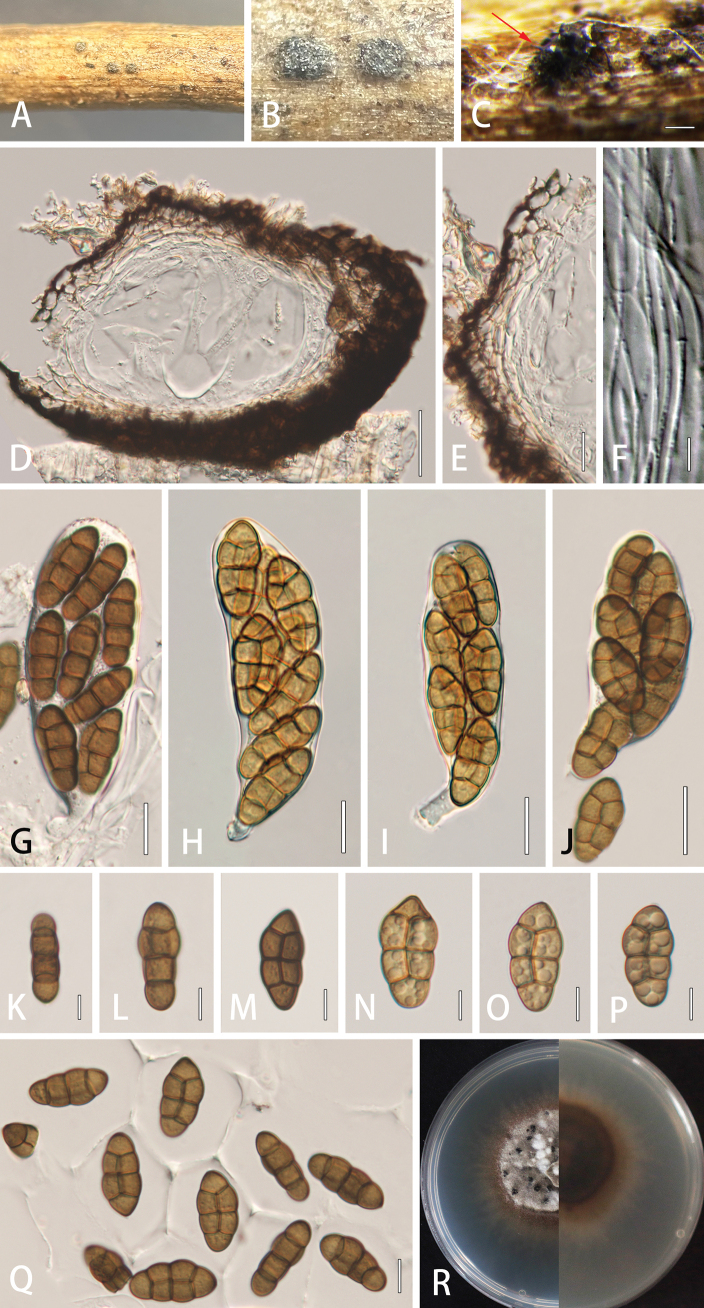
*Comoclathrisxanthoceratis* (HMJAU 64846, holotype) **A–C** appearance of ascomata on host substrate **D** vertical section of ascoma **E** peridium **F** pseudoparaphyses **G–J** asci (**H, I**) asci production from the sterile condition) **K–Q** ascospores **R** culture characteristics on PDA after three weeks at 25 °C (black dots indicate the sexual reproduction in culture condition). Scale bars: 200 µm (**C**); 50 µm (**D**); 20 µm (**G–J**); 10 µm (**E, K–Q**); 5 µm (**F**).

## ﻿Discussion

In this study, we described two new *Comoclathris* species from China, based on morphological and multi-locus phylogenic analyses. The identifying morphological features of *Comoclathris* include operculate perithecia and muriform, asymmetrical, strongly divided ascospores (Shoemaker and Babcock 1992; Wanasinghe et al. 2018). Phylogenetic analyses, based on four combined loci (ITS, LSU, SSU and *rpb*2), as well as morphological characters, are important for the identification of *Comoclathris* species (Table 2). The phylogeny presented here is similar to previous studies (Thambugala et al. 2017; Wijayawardene et al. 2017; Wanasinghe et al. 2018; Brahmanage et al. 2020; Hongsanan et al. 2020), demonstrating a robust backbone tree in this study.

**Table 2.** Synopsis of *Comoclathris* species with the newly-introduced species in bold.

Taxa	Sexual Morph			Asexual morph		Reference
	Ascomata	Asci	Ascospores	Conidiomata	Conidia	
*Comoclathrisantarctica*	339 (± 103) × 299 (± 97) µm, separate or in groups, dark brown to almost black, strongly enclosed in aerial hyphae, ovoid to spherical, without distinct ostiole, neck very short, operculum semi-spherical, flattened; perithecial hyphae dark; wall of 2–3 cell layers.	72–84 × 18–26 µm, mostly 8-spored, immature asci shorter (~ 60 µm), cylindrical to clavate, bitunicate with a rounded apex.	31 × 13.5 µm, lanceolate to ovoid, clavate, yellow to pale brown, elongated, asymmetrical with a blunt apex, muriform, with 6–8 transvers septa, apical cell not divided.	Undetermined		(Crous et al. 2021)
*C.arrhenatheri*	100–150 × 80–120 μm, solitary, scattered or aggregated in small groups, immersed to erumpent, black, elongate, subglobose, covered with pale to dark brown setae, without a distinct ostiole.	65–95 × 18.5–25 μm, 8-spored, cylindrical-clavate, short pedicellate, apically rounded, with an ocular chamber.	16.5–22 × 7.7–10.2 μm, 1–2 seriate, partially overlapping initially yellowish, 1-septate, becoming yellow to pale brown and muriform, with 4 transverse septa and 2–3 vertical septa.	Undetermined		(Thambugala et al. 2017)
***C.clematidis***	**150–230 × 120–150 μm solitary, scattered or aggregated in small groups, immersed to erumpent, subglobose, elongated, black, without a distinct ostiole.**	**114–174 × 27–43 μm, 8-spored, cylindrical-clavate, short pedicellate, apically rounded, with an ocular chamber.**	**22–39 × 8–21 μm, 1–2 seriate, partially overlapping, broadly fusiform, initially 3 septate and yellowish, becoming brown, muriform, with 3 transversely septa and a vertical septum, with a thick mucilaginous sheath.**	**Undetermined**		**This study**
*C.compressa*	200–520 × 150–320 µm, scattered, immersed, sub-epidermal, later superficial, depressed globose, with smooth, straight to bent, tapered, brown hairs.	80–120 × 20–30 µm, numerous, saccate, with tetraserlate to biseriate spores.	24–29 × 10–14 μm, fusoid, straight, transversely 3-septate, with 1 longitudinal septum in central cells, dark reddish-brown, with guttules, smooth, with a uniform sheath 2–3 μm wide.	Undetermined		(Shoemaker et al. 1992)
*C.europaeae*	240–250 × 145–165 µm, solitary, scattered, semi-immersed to slightly erumpent, dark brown to black, globose to subglobose, without a distinct ostiole.	60–70 × 15–18 µm, 8-spored, cylindrical-clavate, pedicellate, apex rounded, with an indistinct ocular chamber.	20–22 × 11–13 µm, uni-to biseriate, partially overlapping, muriform, brown, transversely septate or muriform, with 7 transverse septa.	Undetermined		(Brahmanage et al. 2020)
*C.flammulae*	105–130 × 80–90 µm, solitary or aggregated, immersed, globose to subglobose, dark brown to black, without a distinct ostiole.	50–55 × 13–17 µm, 8-spored, cylindrical-clavate, short pedicellate, rounded at the apex, with an indistinct ocular chamber.	16–22 × 10–16 µm, overlapping uni-to biseriate, yellowish-brown when immature, becoming dark brown at maturity, clavate, with acute ends, muriform, with 6 transverse septa, 1–2 longitudinal septa.	Undetermined		(Brahmanage et al. 2020)
*C.galatellae*	200–550 × 230–340 μm, immersed, erumpent to superficial, broadly to narrowly oblong and flattened, ostiolate.	50–90 × 14–17 µm, 8-spored, cylindrical to clavate, with furcate pedicel and minute ocular chamber.	20–30 × 6–8 µm, uni-seriate or partially overlapping, mostly ellipsoidal, brown or pale brown, muriform, 2–4 transverse septa, 1–2 longitudinal septa, without sheath.	None	2–4 × 1–2 µm, oval to ellipsoid, hyaline, aseptate, guttulate.	(Hongsanan et al. 2020)
*C.italica*	180–240 × 200–250 µm, semi-immersed to erumpent, solitary, scattered, broadly oblong to flattened, dark brown to black, coriaceous, cupulate when dry.	100–120 × 30–35 µm, 8-spored, clavate, short pedicellate, thick-walled at the apex, with a minute ocular chamber.	30–35 × 10–15 µm, overlapping 1–3 seriate, initially 1 septate and hyaline, becoming brown at maturity, muriform, mostly ellipsoidal, 6–8 transversely septate, with 1–2 vertical septa.	Undetermined		(Thambugala et al. 2017)
*C.lini*	260–290 × 300–350 µm, superficial, solitary, scattered, broadly oblong and flattened, dark brown to black, coriaceous, cupulate when dry, ostiolate.	110–130 × 15–25 µm, 8-spored, cylindrical to cylindrical-clavate, pedicellate, thick walled at the apex, with a minute ocular chamber.	20–25 × 10–12 µm, overlapping, initially hyaline, becoming brown at maturity; mostly ellipsoidal, with upper part widest, muriform, with 4–6 transverse septa and 4–6 vertical septa.	Undetermined		(Wanasinghe et al. 2015)
*C.lonicerae*	370–485 × 255–360 µm, solitary or aggregated, scattered, semi-immersed to erumpent, globose to subglobose, dark brown to black, without a distinct ostiole.	180–192 × 60–74 µm, 8-spored, broadly cylindrical to cylindrical clavate, short pedicellate, rounded at the apex, with an indistinct, shallow ocular chamber.	55–70 × 20–30 µm, overlapping uni or biseriate, yellowish-brown, transversely septate or muriform, with 3–5 transverse septa, 1–2 longitudinal septa, with rounded ends.	Undetermined		(Brahmanage et al. 2020)
*C.permunda*	150–200 × 150–200 µm, semi-immersed to erumpent, solitary, scattered, broadly oblong to flattened, dark brown to black, coriaceous, cupulate when dry, with brown to reddishbrown, setae.	90–110 × 19–22 µm, 8-spored, cylindrical-clavate, with a 20–30 µm long pedicel, thick-walled at the apex, with a minute ocular chamber.	22–28 × 9–12 µm, overlapping 1–2-seriate, muriform, mostly ellipsoidal, 2–4 transversely septate, with 1–2 vertical septa, initially hyaline, becoming golden brown at maturity, surrounded by a thick, hyaline, mucilaginous sheath.	Undetermined		(Thambugala et al. 2017)
*C.pimpinellae*	155–135 × 88–95 µm, solitary or aggregated, semi-immersed or rarely somewhat superficial, globose to subglobose, dark brown to black.	58–75 × 14–16 µm, 8-spored, cylindrical-clavate, short-pedicellate, rounded at the apex, with indistinct, shallow, ocular chamber.	14–16 × 5–8 µm, overlapping biseriate, yellow to light brown, transversely septate or muriform, with 3 transverse septa, central segments with 2 longitudinal septa, end segments with 2 angular septa, surrounded by a thick, hyaline, a mucilaginous sheath.	Undetermined		(Li et al. 2016)
*C.rosae*	120–150 × 175–200 µm diam., immersed to erumpent, globose or subglobose, dark brown to black, coriaceous.	70–110 × 15–30 µm, 8-spored, cylindrical-clavate to clavate, pedicellate, thick-walled at the apex, with minute ocular chamber.	20–30 × 8–15 µm, overlapping 1–2 seriate, mostly ellipsoidal, muriform, 4–7 transversely septate, with 1–2 vertical septa, conically rounded at both ends.	Undetermined		(Wanasinghe et al. 2018)
*C.rosarum*	200–300 × 300–400 µm diam., immersed to erumpent, globose or subglobose, dark brown to black, coriaceous.	150–200 × 35–50 µm, 8-spored, clavate, pedicellate, thick-walled at the apex, with minute ocular chamber.	40–60 × 20–25 µm, overlapping 1–2 seriate, mostly ellipsoidal, muriform, 6–7 transversely septate, with 2–4 vertical septa, deeply constricted at the middle septum.	Undetermined		(Wanasinghe et al. 2018)
*C.rosigena*	180–220 × 300–400 µm, immersed to erumpent, globose or subglobose, dark brown to black, coriaceous.	150–180 × 45–60 µm, 8-spored, cylindrical-clavate to clavate, pedicellate, thick-walled at the apex, with minute ocular chamber.	40–60 × 16–24 µm, overlapping biseriate, mostly ellipsoidal, muriform, 5–7 transversely septate, with 1 vertical septum, slightly constricted at the middle septum.	Undetermined		(Wanasinghe et al. 2018)
*C.sedi*	200–250 × 290–350 μm, scattered or aggregated on the host stem, subglobose or nearly globose, superficial, coriaceous, brown to blackish-brown with a blunt ostiole.	80–110 × 16–18 μm, 8-spored, cylindrical to cylindrical-clavate, with a short knob–like pedicel and indistinct shallow ocular chamber.	19–20 × 8–10 μm, 1–2 overlapping seriate, fusiform, muriform, with 4–5 transverse septa and 1–2 longitudinal septa, not constricted at the septa.	Undetermined		(Ariyawansa et al. 2015)
*C.spartii*	Up to 200 μm diam., solitary, scattered or aggregated in small groups, immersed in host tissue, dark brown to black, globose to subglobose, without a distinct ostiole.	100–180 × 23–28 μm, 8-spored cylindrical-clavate, stipitate, apex rounded, with a small apical chamber.	25–34 × 9–14.5 μm, uni- to biseriate in asci, muriform, yellow to pale brown, broadly fusiform, with obtuse ends, constricted at the primary septum, surrounded by a mucilaginous sheath.	Undetermined		(Crous et al. 2014)
*C.typhicola*	350–400 µm diam. Ostiole 100–125 µm diam.	100–125 × 25–30 µm, numerous, clavate, hyaline.	45–50 × 10–12.5 µm, muriform, oval to cylindrical, straight, rounded at one end, slightly tapered at the other, hyaline when immature, light yellow to yellow.	Undetermined		(Adamska et al. 2012)
***C.xanthoceratis***	**147–221 × 114–130 µm, solitary, scattered or aggregated in small groups, , immersed to semi-immersed, subglobose, black, elongated, covered with dark brown setae, without a distinct ostiole.**	**99–165 × 36–48 μm, 8-spored, bitunicate, fissitunicate, clavate, short pedicellate, apically rounded, with an ocular chamber.**	**23–42 × 9–19 μm, 1–2 seriate, muriform, broadly fusiform, with 3 transverse septa and a vertical septum in second and third cells, brown to dark brown, with obtuse ends, smooth-walled, with a thick mucilaginous sheath.**	**Undetermined**		**This study**

In the phylogenetic analyses, many species appear to be conspecific and their phylogenetic placement remains to be resolved. It implied that the concept of subdivision, based on molecular phylogeny alone, has been inaccurate. For example, Wanasinghe et al. (2015) introduced *C.lini* as a new species, although *C.lini* grouped in a well-supported clade with *C.sedi* (100% ML and 1.00 BPP). *Comoclathrislini* is different from *C.sedi* in having comparatively larger asci and different ascospore septa (4–6 transverse septa, 4–6 longitudinal septa vs. 4–5 transverse septa, 1–2 longitudinal septa). In our study, the base pair differences amongst ITS, LSU and *rpb*2 of *Comoclathrisclematidis* and *C.xanthoceratis* were 0.95% (5/526, no gaps), 0.12% (1/803, no gaps) and 4.69% (34/725, no gaps), respectively. There were no differences in the SSU sequences between the two species. The *rpb*2 can be used as an effective barcode to distinguish *Comoclathris* species including *C.clematidis* and *C.xanthoceratis* as it is phylogenetically informative and reflects interspecific relationships (Woudenberg et al. 2013; Ariyawansa et al. 2015; Thambugala et al. 2017; Wanasinghe et al. 2018; Brahmanage et al. 2020). Thus, we recommend using morphological characters coupled with molecular phylogeny to delineate *Comoclathris*, especially including *rpb*2 marker as a protein-coding locus.

The host specificity of *Comoclathris* remains unclear. A single *Comoclathris* species can be found colonising more than one host, while various *Comoclathris* species have also been associated with the same host (Ariyawansa et al. 2015; Thambugala et al. 2017; Wanasinghe et al. 2018; Brahmanage et al. 2020). For example, *C.flammulae* and *C.lonicerae* were found on *Coluteaarborescens* (Brahmanage et al. 2020), while *C.rosae*, *C.rosarum* and *C.rosigena* were found on *Rosacanina* (Wanasinghe et al. 2018). Some *Comoclathris* species have been associated with different hosts. *Comoclathrisarrhenatheri* was collected from *Arrhenatherumelatius* and *Dactylisglomerata* (Thambugala et al. 2017, Italy), while *C.flammulae* was collected from *Clematisflammula* and *Coluteaarborescens* in Italy (Brahmanage et al. 2020).

*Comoclathris* members are mostly distributed in the temperate areas (i.e. Greece, Italy, Netherlands, Russia, Ukraine and USA), while only *C.incompta* (CH-16) and *C.antarctica* (WA0000074564) have been reported in the subtropical and Arctic zones, respectively (Moral et al. 2017; Crous et al. 2021). In this study, *C.clematidis* (CCMJ 13076 and CCMJ 13077) was collected from *Clematis* species (Ranunculaceae) in Kunming City, which is located in the subtropical region. *Comoclathrisxanthoceratis* (CCMJ 13078 and CCMJ 13079) was isolated from *Xanthocerassorbifolium* (Sapindaceae) in Changchun, Jilin Province (temperate zone), which is consistent with many previous studies (Woudenberg et al. 2013; Ariyawansa et al. 2015; Hyde et al. 2016; Li et al. 2016; Thambugala et al. 2017; Wijayawardene et al. 2017; Wanasinghe et al. 2018; Brahmanage et al. 2020; Hongsanan et al. 2020). This study also extends the knowledge of the host range and geographic distribution of *Comoclathris* species.

## Supplementary Material

XML Treatment for Comoclathris clematidis

XML Treatment for Comoclathris xanthoceratis

## ﻿Additional information

### Author ORCIDs

Rong Xu https://orcid.org/0000-0002-7744-6321

Wenxin Su https://orcid.org/0000-0002-5470-5853

Yang Wang https://orcid.org/0000-0002-5899-3987

Shangqing Tian https://orcid.org/0000-0003-4758-3023

Yu Li https://orcid.org/0000-0003-4966-701X

Chayanard Phukhamsakda https://orcid.org/0000-0002-1033-937X

### Data availability

All of the data that support the findings of this study are available in the main text or supplementary information.

## Supplementary materials

Supplementary material 1 Phylogram generated from maximum likelihood analysis based on combined ITS, LSU, SSU, and rpb2 sequnence data Data type pdf https://binary.pensoft.net/file/966746This dataset is made available under the Open Database License (http://opendatacommons.org/licenses/odbl/1.0/). The Open Database License (ODbL) is a license agreement intended to allow users to freely share, modify, and use this Dataset while maintaining this same freedom for others, provided that the original source and author(s) are credited.Rong Xu, Wenxin Su, Yang Wang, Shangqing Tian, Yu Li, Chayanard Phukhamsakda
