# Supplementary figures and images for: ﻿Morphological characteristics and phylogenetic evidence reveal two new species and the first report of Comoclathris (Pleosporaceae, Pleosporales) on dicotyledonous plants from China
Source: MycoKeys. 2024 Jan 12;101:95–112. doi: 10.3897/mycokeys.101.113040 (PMC10799302; doi:10.3897/mycokeys.101.113040)

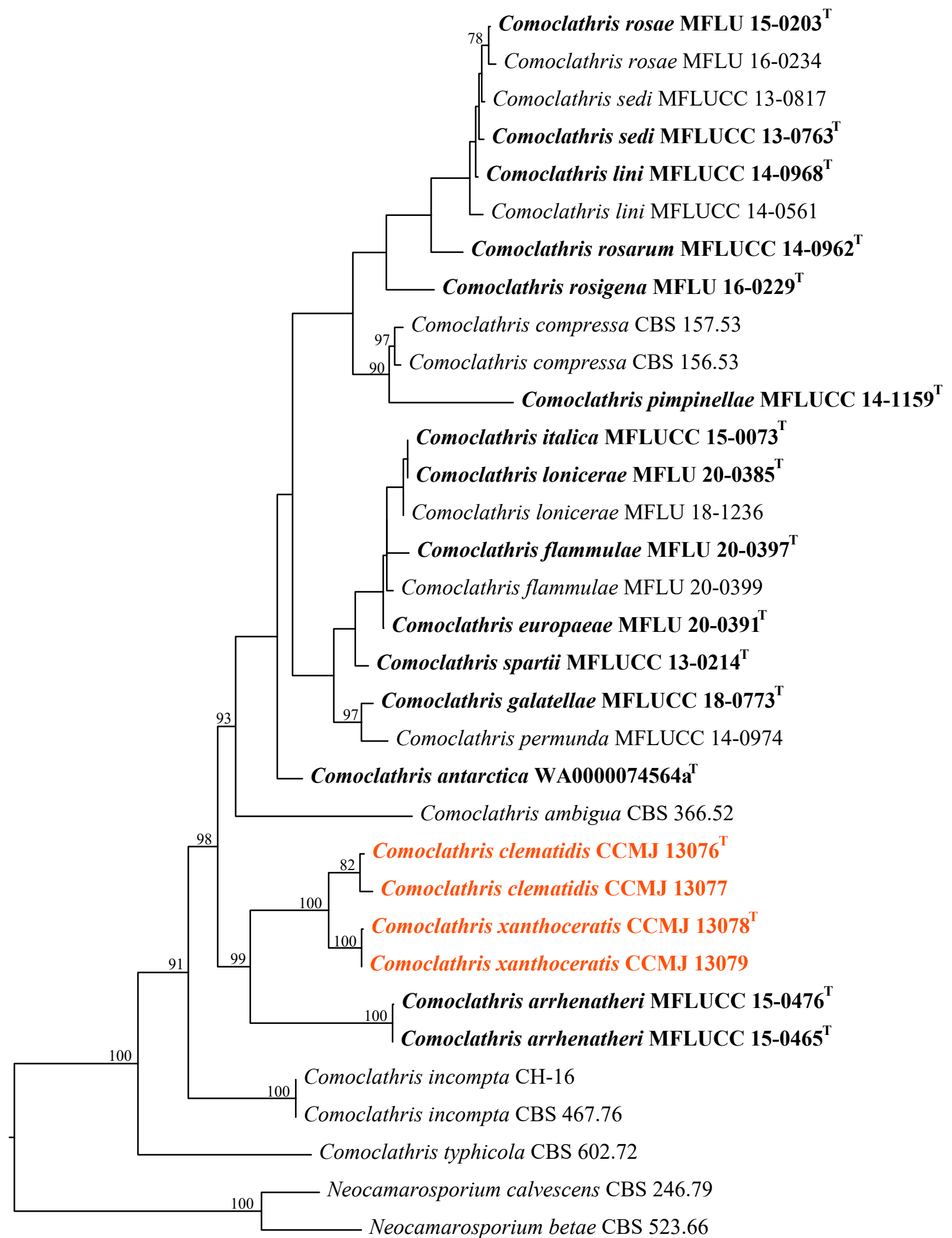

Supplement: Supplementary material 1 — Phylogram generated from maximum likelihood analysis based on combined ITS, LSU, SSU, and rpb2 sequnence data [file mycokeys-101-095-s001.pdf]
